# 

**Published:** 1892-01

**Authors:** 


### INDEX TO VOLUME XLVI.

COMMUNICATIONS. PAGE

Anæsthetics, Practical, by W. E. Walker, D.D.S. ............ 315
Antiseptics, Use of, for Sterilizing Cavities before Filling, by H. A. Smith, D.D.S....... 127
Antizymotics, or Agents which Prevent Fermentation and Putrefaction, by N. S. Hoff, D.D.S.............................. 569
Application of Vapor under pressure for Diseased Tissue, by Dr. Francis Peabody ..... 494
Address by President, E. G. Betty, D.D.S..................... 47
Address by President, L. E. Custer, D.D.S. .................... 188
Address by President, Dr. H. K. Lathrop........................ 1
Address of Welcome by Col. S. M. Meek....................... 307
Annual Address by Dr. D. B. McHenry ......................... 310
Address, Valedictory, J. J. R. Patrick, D.D.S................. 263
Address, Annual, Dr. Gordon White............................ 465
Address, Annual, Dr. W. W. Walker............................ 517
Antrum, Surgery of, by G. S. Junkerman, M.D., D.D.S.... 178
Bacteriology, The Importance of a Knowledge of, to the Dentist, by Carrie M. Stewart, D.D.S........................ 580
Care of Children's Teeth, Dr. S. W. Foster..................... 485
Care of Teeth during Pregnancy and Lactation, by E. H. Raffensperger, D.D.S...... 48
Combining Business Methods with Professional Methods in Dealing with Our Patients, by C. B. Blackmarr, D. D.S.................................. 586
Combination Fillings, by J. R. Callahan, D.D.S................ 99
Contentment, A Poem, by J. A. Robinson, D.D.S............ 279
Crown and Bridge Work, by Grant Mitchell, D.D.S....... 439
Crown and Bridge Work, Some Practical Points in Regard to Methods in Construction of, by G. W. Melotte, D.D.S. 131
Crown Setting, A New Oxyphosphate for, by W. B. Ames, D.D.S..................... 184
Correcting Irregularities of the Teeth, by Prof. C. S. Case... 27
Decay, Primary Causes of, Dr. Geo. B. Clement................. 312
Decolorization of the Teeth, by A. O. Rawls, D.D.S. .......... 168
Dentition, Various Kinds of, by N. E. Root, D.D.S......... 278
Dentists and Dentistry as seen by Our Patients, by Dr. W. H. Wright........................ 402
Dentistry in the Forties, by L. C. Whiting....................... 583
Diagnosis of Chancre, Prognosis of Treatment, by C. G. Darling, M.D.................... 328
Diagnosis, by J. R. Bell, D.D.S. 416
Difficult Dental Prosthesis, by I. Douglas, D.D.S............. 591
Discretion in the Performance of Dental Operations, by F. W. Sage, D D.S................ 203
Early Extraction of the Sixth Year Molar, Some of the Results of the, by W. B. Conner, D.D.S................... 334
Electricity as a Therapeutic Agent, by Dr. J. S. Marshall, 489
Effect of the Internal Administration of Certain Drugs upon the Tissues of the Oral Cavity, by Dr. J. D. Patterson .............................. 538
Erosion, by W. S. Elliott, M.D., D.D.S............................ 108
Europhen and Trichlor Acetic Acid, by A. W. Harlan...... 533

PAGE

Formation and Care of the Teeth, by Dr. K. L. Cleaves, 384
Grinding Teeth of the Herbivorous Mammalia, by A. H. Thompson, D.D.S............... 522
Gold Fillings, Some of the Causes of Failure in, by Dr. J. H. Allen......... ............ 491
Gutta-Percha as a Root Canal Filling, by Forest G. Eddy, D.M.D.......................... 425
Heredity, by A. O. Rawls, D.D.S........................... 68
Hints from the Office and Laboratory, by Dr. Isaac Douglas........................... 5
Infection, by H. I. Ambler, M.D., D.D.S..................... 413
Immediate Root Filling, by Dr. S. Hubbell................. 394
Irregularities of the Teeth, Correcting, by Prof. C. S. Case.............................. 27
Ichoræmia, by C. R. Butler, M.D., D.D.S.................... 116
Management of Practice, by W. H. Whitslar, D.D.S....... 274
Matrices, by J. E. Cravens, D.D.S.............................. 530
Micro-Organisms of the Mouth by J. H. Linsley, M.D........ 368
Nitrous Oxide in Cardiac Weakness, A Plea for, by Dr. S. P. Sharpe............... 487
New Oxyphosphate for Crown Setting, by W. B. Ames, D.D.S.............................. 184
Overwork, by Dr. Henry Cowie, 31
On Edge, by C. M. Wright, D.D.S.............................. 212
Practical Anæsthetics, by Dr. W. E. Walker................... 315
Pental, the New Anæsthetic, by O. N. Heise, D.D.S......... 161
Permanent Closure of the Jaws, With Report of a Case, by Wm. Knight, M.D., D.D.S., 170
Plea for Nitrous Oxide in Cardiac Weakness, by Dr. S. P. Sharpe........................... 487
Philosophical Ideas in Operative Dentistry, by E. C. Chisholm.............................. 493
Prophylaxis, by J. Taft, D.D.S. 118

PAGE.

Pulp Protection by Cavity Lining, by G. F. Cheney, D.D.S. 396
Pyorrhœa Alveolaris, A Case in Practice, by J. E. Cravens, D.D.S.............................. 519
Relationships, by H. H. Harrison................................ 62
Replanting and Transplanting Teeth, by W. N. Morrison, D.D.S............... .............. 528
Strange Condition, Did Arsenic Produce It? by Dr. G. E. Corbin .......................... 17
Sterilized Sponge in Pulp Capping, by Dr. T. H. Parramore............................... 496
Skin and its Appendages with Special Reference to the Development of the Teeth, by H. M. Fletcher, M.D., D.D.S.............................. 154
Soliloquy of a Plastidule, by Dr. C. M. Wright, D.D.S...... 65
Some of the Causes of Failure in Gold Fillings, by Dr. J. H. Allen.......................... 491
Some of the Results of the Early Extraction of the Sixth Year Molar, by W. B. Conner. D.D.S................... 334
Surgery of the Antrum, by G. S. Junkerman, M.D., D.D.S., 178
Systematic Medication, by Dr. N. S. Hoff........................ 21
Syphilis, Synopsis of a Lecture on, by C. G. Darling........... 255
Syphilitic Disease of the Tongue, by C. G. Darling, M.D.............................. 361
Syphilitic Disease of the Nose, by C. G. Darling, M.D........ 433
Syncope and Asphyxia, by G. H. Wilson, D.D.S............... 429
Teeth, Care of During Pregnancy and Lactation, by E. H. Raffensperger, D.D.S...... 48
Teeth, Treatment of During Pregnancy, by Dr. J. W. VanDoorn........................ 138
Teeth, Decolorization of the, by A. O. Rawls, D.D.S........ 168
Teeth, Replanting and Transplanting, by W. N. Morrison, D.D.S...................... 528

PAGE

Teeth, Formation and Care of the, by Dr. K. L. Cleaves.... 384
Teeth of the Proboscidians, A Brief Study, by W. C. Barrett, M.D., D.D.S.............. 523
Tumors of the Mouth, by J. Taft, D.D.S.................... 270
Use of Gutta-Percha as a Root Canal Filling, by Forest G. Eddy, D.M.D.................... 425
Use of Sterilized Sponge in Pulp Capping, by Dr. T. H. Parramore...................... 496
Various Kinds of Dentition, by N. E. Root, D.D.S......... 278
What Causes Variety and Modifications in the Character of Dental Caries, by G. S. Junkerman, M.D., D.D.S........... 86
What Changes Occur in the Teeth with the Advancement of Age, by V. A. Latham, D.D.S................ 543
The Work and Duties of the Ohio State Dental Society, by W. H. Sedgwick, D.D.S., 151
The World's Columbian Dental Congress......................... 192
The World's Congress Auxiliary of the World's Columbian Exposition................ 450

### PROCEEDINGS.

American Dental Association, 517
Colorado State Dental Association................................ 541
Mississippi State Dental Association, Eighteenth Annual Session... ....... 307, 454
Mississippi Valley Dental Association........................ 222
Michigan Dental Association, Thirty-Sixth Annual Meeting.................................... 1
Michigan Dental Association. Thirty-Seventh Annual Meeting.......................... 597
National Association of Dental Faculties.......................... 501
National Association of Dental Examiners........................ 505
Northern Ohio Dental Association.......................... 286

PAGE

Ohio State Dental Society, Twenty-Fifth Annual Meeting................................ 35
Post Graduate Dental Association.............................. 348
Report on Necrology........... 455
Seventh District Dental Society of Ohio...................... 284
Southern Dental Association, 465
Vermont State Dental Society, 405
Walnut Hills Medical Society, 144
West Virginia State Dental Society.......................... 455

### SELECTIONS.

Ancestry of Chalicotherium... 299
Antisepsis for the Hands...... 145
Artificial Production of Variation in Types.................. 300
An M.D. Self-Qualified......... 300
Bacteria, Practical Use of...... 457
Battle With Germs............. 339
Blackening of the Teeth by Antipyrine...................... 262
Brain Rest and Sleep.......... 296
Boils................................. 557
Burns................................ 350
Character........................... 561
Cocaine Poisioning................ 559
Cocaine, To Dissolve............ 301
Creosote and its Elements...... 509
Dental Law in the District of Columbia......................... 345
Dental Law of Ohio, Amended, 226
Defining a Blush.................. 561
Death of the Inventor of the Hypodermic Syringe......... 464
Doctors and Politics............. 34
Doctor as a Professional Leader, The............................ 551
Effects of Variously Colored Lights on Insane Persons.... 551
Facial Paralysis.................... 556
Gold, Therapeutics of.......... 558
Gum Chewing, The Ethics of, 297
Gluten Foods...................... 547
Hereditary Transmission of Mutilations....................... 512
Higher Professional Standard, A.................................... 146
How Medicines Act............ 299
Human and Animal Blood.... 298
Hypodermic Syringe, Death of the Inventor................. 464

PAGE
Indigestibility of Certain Articles... 510
Keep Your Head Clean... 558
Keeley Treatment, The... 556
Lysol... 91
Method of Bleaching Sponges, 561
The Moral Side... 559
Nausea Following Anæsthesia, 301
Natural Result, The... 554
Neuralgia, Dental... 560
Neuralgias and Neuralgic Affections... 553
Neuralgia, Treatment of by Hypodermic Injections of Cocaine... 512
Nerve Stretching in Trigeminal Neuralgia... 449
On the Teaching of Anatomy to Advanced Medical Students... 287
Overdose of Gold, An... 283
Opium Habit, The... 247
Odontalgia Cured by Ipecac... 542
Oxygen, To Obtain Pure, Rapidly... 98
Oxysulphate of Zinc, Uses for the... 606
Pain and Inflammation of Dental Origin... 294
Patent Medicines in Turkey... 552
Peroxide of Hydrogen, Impurities in Commercial Samples... 92
Pointer, A... 327
Position in Sleep... 609
Practical Use of Bacteria... 457
Ratio of Physicians to Population... 552
Resuscitation After Drowning, 462
Reminders of the Past... 552
Salicylic Acid... 560
Sterilization of Metallic Instruments... 412
Simple Water Test... 562
Tetanus, Cure of, with the Antitoxin Obtained from the Serum of an Immune Animal... 555
Treatment of Disorders of the Teeth during Pregnancy... 513
Treatment of Physical Pain... 596
Tooth Culture... 462
To Stimulate Appetite... 554
To Remove Rust... 557

PAGE
The True Physician... 562
To Obtain Pure Oxygen Rapidly... 98
A Vexed Question... 511
Walking... 555
Water Test, A Simple... 562
Wash for Chapped Hands... 579

COLLEGE COMMENCEMENTS

American College of Dental Surgery... 245
Boston Dental College... 409
Baltimore College of Dental Surgery... 232
Cincinnati College of Medicine and Surgery, Dental Dept... 234
Chicago College of Dental Surgery... 235
Columbian University, Dental Department... 238
German American Dental College... 407
Harvard University, Dental Department... 410
Howard University, Dental Department... 410
Homœpathic Hospital College, Dental Department... 246
Indiana Dental College... 242
Kansas City Dental College... 236
Missouri Dental College... 234
Meharry Medical College, Dental Department... 233
Northwestern University, Dental Department... 247
New York College of Dental Surgery... 243
Ohio College of Dental Surgery, 242
Philadelphia Dental College, 230
Pennsylvania College of Dental Surgery... 239
Royal College of Dental Surgeons of Ontario... 237
Southern Medical College, Dental Department... 233
Tennessee Medical College, Dental Department... 239
United States Dental College, 231
University of California, College of Dentistry... 241
University of Iowa, Dental Department... 237
University of Maryland, Dental Department... 240

PAGE
University of Michigan, College of Dental Surgery....... 410
University of Minnesota, Dental Department................ 411
University of Pennsylvania, Department of Dentistry..... 408
Vanderbilt University, Dental Department..................... 244
Western Dental College of Kansas City...................... 238

EDITORIAL.

A Correction, The World's Columbian Dental Congress 611
American Dental Association, 360
About Dental Colleges........... 353
Aluminum the Metal of the Future.... ........................ 41
Biographical, Dr. Daniel W. Roudebush........................ 461
Bibliographical 93, 94, 150, 305, 611
Columbian Dental Congress.... 563
Educational.......................458, 514
Hymeneal.........................95, 306
Isaac Knapp Dental Coterie, The.................................. 252
Knoxville City Dental Society, 568
Mississippi Valley Dental Association.......................... 96
A New Disc Mandrel............ 97
Ohio State Dental Society...... 564
Ohio State Dental Society, Annual Meeting.................... 516
Ohio Dental Law, Amended... 250
Pan-American Medical Congress .................. 460, 565
Professional, Change of Location................................ 150
Robinson, Dr. J. A............... 259
Seventh District Dental Society of Ohio........................... 201
Southern Dental Association, 358
Tacoma Dental Society......... 412
World's Columbian Exposition......... 45, 148, 303, 459
World's Columbian Dental Congress..............97, 357, 463

PAGE
Washington State Dental Society................................. 411

NOTICES.

American Dental Society of Europe............................ 248
American Medical Association, Section of Dental Surgery.............................. 249
American Dental Association, 351
California State Dental Association.......................... 352
Florida State Dental Association............................. 248
Illinois State Dental Society... 249
Kentucky State Dental Association.......................... 302
National Association of Dental Faculties..................... 351
National Association of Dental Examiners.................. 352
Ohio State Board of Dental Examiners....................... 568
Post Graduate Dental Association of the United States... 95
Pennsylvania and New Jersey State Dental Societies......... 351
State of New York Dental Society.............................. 248
South Carolina State Dental Association...................... 352
West Virginia State Dental Society........................301, 563

CORRESPONDENCE.

The Electric Deposit Plates.... 39
The Shaw Dental Engine........ 41

OBITUARY.

Allen, Dr. John.................... 253
Cameron, Dr. Jos. G............. 202

IN MEMORIAM.

Allen, Dr. John.................... 455
Kingsbury, C. A., M.D., D.D.S., 456

### DIRECTORY OF DENTAL SOCIETIES.

*American Dental Association*—Meets on the first Tuesday of August, 1892, at Niagara Falls. Pres., W. W. Walker, Rec. Sec., Geo. H. Cushing, Chicago.

*Southern Dental Association*—Meets at Harrogate, Tenn., last Tuesday in July, 1892. Prest., Go don White, Nashville; Secretary, M. C. Marshall, Little Rock, Ark.

### STATE DENTAL SOCIETIES.

*Alabama Dental Association*—Meets annually. Next meeting at Montgomery, on the second Tuesday of April, 1892. Pres. Geo. Eubank, Birmingham; Sec. J. H. Allen, Birmingham.

*California State Dental Association*—Third Tuesday in July, 1892. Pres., Thos. Morffew; Rec. Sec., W. A. Knowles, San Francisco; Cor. Sec., L. Van Orden. Next Meeting in San Francisco.

*Colorado State Dental Society*—Meets 2nd Tuesday in June, 1892. Pres., R. B. Weiser, Georgetown. Colorado; Sec., C. Conert, Denver. Next meeting will be held at Colorado Springs.

*Connecticut State Dental Society*—Pres. E. A. Stebbins, Shelburne Falls; Rec. Sec. Geo. A. Maxfield, Holyoke. Annual meeting Oct. 27th and 28th, 1892, at Hartford.

*Dental Society of the State of Maryland and District of Columbia*—Meets annually. Next meeting at Washington City, on the second Tuesday in October, 1892. Pres. B. F. Coy, of Baltimore: Sec. M. W. Foster, M. D.

*Georgia State Dental Society*—Meets second Tuesday in July, 1892, at Rome Pres. W. G. Browne; Rec. Sec. S. H. McKee; Cor. Sec. L. D. Carpenter, Atlanta.

*Illinois State Dental Society*—Meets at Springfield, second Tuesday in May, 1892. Pres., T. W. Pritchett, Chicago; Sec., G. Newkirk, Chicago.

*Indiana State Dental Society*—Meets next at Maxinkukee, on the last Tuesday of June, 1892. Pres., E. J. Church, LaPorte, Ind.; Sec., G. E. Hunt, Indianapolis.

*Iowa State Dental Society*—Meets annually. Next meeting in Ottumwa, on the first Tuesday in May, 1892. Pres. S. E. Hatch, Sioux City; Rec. Sec. G. W. Miller, Des Moines.

*Kentucky State Dental Association*—Meets annually, first Tuesday in June, 1892. Next meeting in Louisville. Pres., H. B. Tileston, Louisville; Sec., J. H. Baldwin, Louisville.

*Kansas State Dental Association*—Pres. W. M. Shirley, Hiawatha; Sec. C. B. Gunn, Leavenworth. Next meeting will be held at Topeka, April 30th, 1892.

*Maine Dental Society*—Pres. G. W. Stoddard: Sec. C. E. Bryant, Pittsfield. Meets on the third Tuesday in July, 1892. Place of meeting not decided.

*Maryland State Dental Association*—Meets annually in December. Pres. J. Emory Scott; Rec. Sec. W. W. Dunbracco; Cor. Sec.

*Massachusetts Dental Society*—Meets Semi-Annually in Boston, June 5th, 6th and 7th, 1892. Pres. Geo. F. Eames, Boston; Rec. Sec. E. O. Kinsman, Cambridge

*Michigan State Dental Association*—Meets annually. Next meeting at Saginaw, Tuesday, June 7th, 1892. Pres., H. C. Corns: Detroit; Sec., J. Ward House, Grand Rapids. Treas. Geo. H. Mosher, Jackson.

*Mississippi State Dental Association*—Meets July 18th, at Biloxi, 1892, during Camp Meeting. Pres., R. K. Luckie, Holly Springs; Sec., R. H. Hight, Grenada.

*Minnesota State Society*—Pres. W. C. Merrill, Albert Lee; Sec. L. D. Leoard, Minneapolis. Meets in Minneapolis, July 13th, 1892.

*Missouri Dental Association*—Meets at Clinton, Mo., July 5th to 9th, inclusive, 1892, Pres., Geo. L. Shepard, Sedalia; Sec., W. M. Carter, Sedalia.

*Nebraska State Dental Society*—Meets annually. Next meeting third Tuesday in May 1892, at Beatrice; Pres. J. J. Willey, Wahoo; Sec. C. R. Tefft, Lincoln.

*New Jersey State Dental Society*—Meets annually. Place and time of meeting to be decided later. Pres. B. F. Luckey, Paterson; Sec. C. A. Meeker, Newark.

*New Hampshire Dental Society*—Meets Annually. Next meeting will be held in Concord, N. H., second Wednesday in June, 1892. Pres. W. R. Blackstone, Manchester; Sec. Burton C. Russell, Keene.

*North Carolina State Dental Society*—Meets in Morehead City, August 11, 1892. Pres. H. C. Hearing, Concord; Sec., C. A. Rominger, Reidsville.

*North Dakota Dental Society*—Meets July 26, 1892. Pres. S. P. Johnson, Grand Forks; Sec. S. J. Neill, Fargo.

*Ohio State Dental Society*—Meets annually. Next meeting at Columbus, first Tuesday of December, 1892. Pres. J. R. Callahan, Cincinnati; Sec. Otto Arnold, Columbus.

*Pennsylvania State Dental Society*—Time and place not yet decided of meeting. Pres. Louis Jack, Philadelphia; Cor. Sec. H. Newton Young, Wilkesbarre.

*Rhode Island Dental Association*—Pres., D. F. Keefe, Providence; Sec., W. H. Carpenter, Providence. Meets quarterly, second Tuesday in July, October, January and April. Annual Meeting second Tuesday in July, at Providence.

*South Carolina State Dental Association*—Meets annually. Pres. J. T. Calvert, Spartanburg; Cor. Sec. A. T. Peete, Branchville. Next meeting at Rock Hill, S. C., third Tuesday in May, 1892.

*Texas State Dental Association*—Meets in Ft. Worth, fourth Tuesday in May, 1892. Pres., J. H. Grant, Austin; Sec., C. B. Lewis, Dallas.

*Tennessee Dental Association*—Meets Annually. Next meeting at Columbia, first Tuesday in July 1892. Pres. W. H. Morgan, Nashville; Rec. Sec. A. G. White, Springfield; Cor. Sec. E. G. Grant, Columbia.

*The Dental Society of the State of New York*—Meets annually on the second Wednesday in May. Next session at Albany, May 8, 1892. Pres. W. W. Walker, New York; Rec. Sec., F. T. Van Woert, Brooklyn; Cor. Sec., R. Ottolengui, New York.

*Vermont State Dental Society*—Meets annually. Next meeting at Burlington, third Wednesday in March 1892. Pres. Dr. W. S. Curtis, West Randolph; Sec. Thos. Mound, Rutland.

*Virginia State Dental Association*—Meets in Charlottsville, Tuesday, August 19, 1892. Pres. J. Hall Moore; Sec. L. M. Cowarden, Richmond.

*Wisconsin State Dental Society*—Meets annually. Next meeting at Milwaukee, third Tuesday in July, 1892. Pres. E. C. French, Ean Claire; Sec. C. A. Southwell, Milwaukee.

*West Virginia State Society.*—Meets at Wheeling, W. Va., January 7th, 1892. Pres. H. H. Harrison, Wheeling; Sec. Geo. I. Keener, Morgantown.

### LOCAL SOCIETIES.

*American Academy of Dental Science*—Meets annually in Boston, Mass., on the first Wednesday in October, 1892. Pres. J. H. Batchelder; Rec. Sec. E. E. Hopkins; Cor. Sec. E. B. Hitchcock, Boston.

*Brooklyn Dental Society and Second District Society United*—Meets quarterly—Pres. T. H. Holly; Rec. Sec. A. N. Russell, Brooklyn.

*Central Dental Association of Northern New Jersey*—Meets bi-monthly; and annually in February. Pres. C. W. F. Holbrook, Newark; Sec. A. S. Hawley, Newark.

*Chicago Dental Society*—Meets monthly. Pres. D. M. Cattell; Sec. L. S. Davis, Cor Sec. T. L. Gilmer.

*Chicago Dental Club*—Meets on the fourth Monday of each month. Pres. A. B. Freeman; Sec. C. Stoddard Smith.

*Cleveland Dental Society.*—Meets the first Monday of each month. Pres. S. B. Dewey; Sec. Martha J. Robinson.

*Connecticut Valley Dental Society*—Meets 27 and 28 of Oct. 1892, at Savin Rock Conn. Pres. C. T. Stockwell, Springfield, Mass. Sec. A. M. Ross, Chicopee.

*Detroit Dental Society*—Meets monthly, Pres. E. C. Moore, Sec. Wm. Cleland.

*Eighth District Dental Society, N. Y.*—Annual meeting third Tuesday in April 1892, in Buffalo. Pres. F. W. Low, Buffalo; Sec. W. A. Barrows, Buffalo.

*East Tennessee Dental Association*—Meets annually. Next meeting will be held at Chattanooga, Tenn., on the first Tuesday of Aug., 1892. Pres. R. A. B. Noyers; Sec. S. N. Prothro.

*First District Dental Society of Illinois*—Next meeting will be held in Peoria, first Tuesday in September, 1892. Pres E. K. Blair, Waverly; Sec. W. O. Butter, La Harpe.

*Mississippi Valley Dental Society*—Meets annually at Cincinnati, on first Wednesday in March, 1892. Pres. L. E. Custer, Dayton; Sec. H. T. Smith, Cincinnati.

*New England Dental Society*—Meets annually. Next meeting first Thursday in Oct., 1892. Place of meeting to be decided later. Pres. J. F. Adams, Worcester, N. H.; Sec. E. O. Kinsman, Cambridge, Mass.

*Northern Illinois Dental Society*.—Meets annually. Next meeting at Freeport, October 15th, 1892. Pres. G. W. Dennis, La Salle. Sec. T. W. Beckwith.

*Northern Ohio Dental Association*—Meets annually. Next meeting at Cleveland, O. on the second Tuesday in May, 1892. Pres. J. F. Siddell, Oberlin; Rec. Sec. H. G. Hustice, Oberlin; Cor. Sec. R. H. Barnes, Cleveland, O.

*Northwestern Dental Association* (embraces portions of Dakota, Minnesota and Manitoba) meets annually. Next meeting at Detroit, Dak. on the fourth Tuesday in July, 1892. Pres. J. W. Cloes, Jamestown, Dak. Sec. S. J. Hill, Fargo, Dak.

*Odontological Society of Pennsylvania*—Meets on the first Saturday evening of each month, at 8 o'clock p. m., at 13th and Arch sts; Pres. Jas. Truman, Rec. Sec. A. W. Deane.

*Odontological Society of New York City*—Meets the third Tuesday of each month. Pres. Wm. Jarvie; Rec. Sec. E. T. Payne.

*Odontological Society of Cincinnati*—Meets monthly. Pres. C. M, Wright; Sec. W. M. Williams.

*Odontological Society of Chicago*—Meets monthly. Pres. G. Newkirk; Sec. E. Noyes.

*Ohio Valley Dental Society*.—Meets quarterly. Next meeting first Monday in July, 1891, at Stuebenville, O. Pres. N. H. Harrison, Cadiz; Sec'y F. S. Maxwell, Stuebenville, O.

*Pennsylvania Association of Dental Surgeons*—Meets second Tuesday of each month; Pres. H. E. Roberts; Cor. Sec. Theo. F. Chupein.

*Pittsburg Dental Society*—Meets the last Tuesday Evening of each month. Pres. J. B. Williams, Homestead; Sec. N. L. Reinecke.

*Fifth District Dental Society of N. Y.*—Meets semi-annually. Next meeting in Syracuse, October, 24-26. Pres. R. F. Jones, Utica; Sec. T. B. Mason, Phoenix.

*San Francisco Dental Society*—Meets the second Monday evening of each month. Pres. W. A. Knowles; Sec. Chas. Morffew; Treas. J. J. Birge.

*Sixth District Dental Society of N. Y.*—Meets semi-annually. Next meeting at Syracuse, Oct. 24-26, 1892. Pres. Wm. H. Hall, Binghamton; Sec. Myron D. Jewell, Richfield Springs.

*Seventh District Dental Society of Ohio*—Meets annually, Third Tuesday in May. 1892. Next meeting at Washington C. H. Pres., O. N. Heise, Cincinnati; Sec., J. R. Callahan, Cincinnati.

*Southern Minnesota Dental Society*—Next meeting in Mankato, third Tuesday in April, 1892. Pres. C. D. Snow, Mankato,; Sec. W. D. James, Tracy.

*St. Louis Dental Society*—Meets first and third Tuesday in each month. Pres. John G. Harper; Cor. Sec. Emma E. Chase; Rec. Sec. DeCoursey Lindsley.

*The Minneapolis Dental Society Monthly*—Pres. E. F. Clark, Sec. E. J. Morrison.

*Washington Dental Society*—Meets second Tuesday of each month. Pres. R. Finley Hunt; Rec. Sec. H. M. Schooly.

*First District Dental Society of the State of New York*—Meets annually. Pres. A. L. Northrop; Sec. J. E. Dexter, New York.

*Washington City Dental Society*—Pres. M. B. Noble; Sec. Wm. Donally. Meets monthly.

### CONTENTS.

COMMUNICATIONS.

The Thirty-sixth Annual Meeting of the Michigan Dental Association........ 1

Over Work........ 31

PROCEEDINGS.

Twenty-fifth Annual Meeting of the Ohio State Dental Society....... 35

CORRESPONDENCE.

The Electro-Deposit Plates........ 39

EDITORIAL.

Aluminum—The Metal of the Future........ 41
The World's Columbian Exposition........ 45

### THE DENTAL REGISTER,

**A MONTHLY MAGAZINE DEVOTED TO THE INTERESTS OF THE**

**DENTAL PROFESSION.**

*Terms Reduced to* $2.00 *Per Year. Single Copies,* 25 *Cents.*

**EDITED BY PROF. J. TAFT, D.D.S.**

—AND—

**Published by SAM'L A. CROCKER & CO., Ohio Dental and Surgical Depot.**

The volume commences in January and closes in December.

The REGISTER being issued monthly is always fresh, therefore Indispensable to the practitioner who desires to keep posted up with the new inventions and improvements which are daily developed. Neither expense nor effort will be spared to give value and variety to its contents. Articles will be illustrated with well executed engravings whenever they will add to the interest or assist to explanation.

Its extensive circulation and frequency of issue make it one of the BEST DENTAL ADVERTISING MEDIUMS in the United States.

All subscriptions must begin with January or July.

Local or special notices, five lines or less, will be inserted for $3 each insertion; over five lines, 50 cts. per line.

All advertising bills payable in advance, or at furthest, after the issue of the first number containing the advertisement. All transient advertisements to be paid for in advance.

☞CONTRIBUTIONS SOLICITED.

Exclusively Editorial Communications should be addressed to the Editor.

The latest number of the REGISTER may be ordered with or without other goods at any time, and all letters should be addressed to

SAM'L A. CROCKER & CO., Ohio Dental and Surgical Depot, Cincinnati, O.

